# Attention and Motor Learning in Adult Patients with Neurofibromatosis Type 1

**DOI:** 10.1177/10870547211012035

**Published:** 2021-05-12

**Authors:** Jesminne Castricum, Joke H. M. Tulen, Walter Taal, André B. Rietman, Ype Elgersma

**Affiliations:** 1 Erasmus University Medical Center, Rotterdam, The Netherlands; 2 The ENCORE Expertise Center for Neurodevelopmental Disorders, Erasmus MC, Rotterdam, the Netherlands; 3 Erasmus University Medical Center Sophia Children’s Hospital, Rotterdam, The Netherlands

**Keywords:** neurofibromatosis type 1, adult, neuropsychological functioning, motor performance, cognition

## Abstract

**Objective:**

Neurofibromatosis type 1 (NF1) is an autosomal dominant genetic disorder that is associated with cognitive disabilities, including attention and motor learning problems. These disabilities have been extensively studied in children with NF1 but limited studies have been performed in adults.

**Method:**

Attention, motor learning and intellectual performance were studied with neuropsychological tasks in 32 adults with NF1 and 32 controls.

**Results:**

The NF1 and control group performed similarly on attention and motor learning tasks, although controls had shorter reaction times than adults with NF1 during the motor learning task (*t*[60] = −2.20, *p* = .03). Measures of attention or motor learning were not significantly associated with reduced intellectual performance in NF1.

**Conclusion:**

In contrast to many studies in children with NF1, our findings did not provide evidence for presence of attention or motor learning problems in adults with NF1 in neuropsychological tasks. Our observations may be of clinical importance to determine treatment focus in adults with NF1.

## Introduction

Neurofibromatosis type 1 (NF1) is an autosomal dominant genetic disorder caused by a heterozygous loss-of-function mutation in the NF1 gene (Cawthon et al., 1990). NF1 is frequently associated with cognitive disabilities, in addition to the characteristic somatic features (Ferner, 2007). These cognitive disabilities include reduced intelligence and deficits in attention and motor learning, which have been well-documented and assessed more extensively in children with NF1 (Chaix et al., 2018; Hyman et al., 2005; Krab, Aarsen, et al., 2008, 2011; Ottenhoff et al., 2020; Rietman et al., 2017; Van der Vaart et al., 2016). Cognitive impairment has been shown to relate to decreased quality of life in children and adolescents with NF1 (Varni et al., 2019). Although attention and motor learning deficits have been extensively studied in children with NF1, limited studies have focused on adults with NF1.

Attention is the most frequently affected ability in children with NF1, next to learning disabilities and motor problems, with observed attention deficits in 33% to 50% of the children and with an overrepresentation of ADHD (Templer et al., 2012). The domain of attention is most often studied in NF1 cognitive clinical trials (Walsh et al., 2016) with wide variability in the use of tools to measure attention. To our knowledge, only a few other studies have investigated attention in adults with NF1 with contradictory findings (Descheemaeker et al., 2013; Ferner et al., 1996; Mautner et al., 2015; Pavol et al., 2006; Zoller et al.,1997). In twenty adults with NF1, impairments in attention were shown using a neuropsychological test battery (Zoller et al., 1997), consistent with findings in children with NF1. Moreover, Ferner et al. (Ferner et al., 1996) observed impaired attention in a large cohort of 103 NF1 patients with an age range of 6 to 75 years, although differences between children and adults were not investigated separately. More recent studies in twenty adults with NF1, showed no deficits in attention, including selective and sustained attention (Descheemaeker et al., 2013) or visual attention (Pavol et al., 2006).

Attention problems may be associated with difficulties in motor learning observed in children with NF1 (Krab et al., 2011). Children with ADHD showed a high prevalence of disabilities in fine motor skills (Mokobane et al., 2019). In addition, previous studies in children with NF1 showed disabilities in fine motor skills, motor speed, and motor performance (Iannuzzi et al., 2016; Johnson et al., 2010; Krab et al., 2011; Rietman et al., 2017). Neuroimaging studies in children with NF1 showed an association between deficits in cognitive deficits including motor skills and cerebral physiopathology, although the exact link remains unclear (Baudou et al., 2019, 2020). Motor problems have also been shown in 44 young adults with NF1 with disabilities in fine motor skills (Feldmann et al., 2003) and in 21 adults with NF1 with reduced voluntary muscle force (Souza et al., 2009). One study investigated motor skill learning in nine adults with NF1 by using the sequential finger-tapping task and found that motor learning was affected (Zimerman et al., 2015). In contrast, an older study observed no specific problems in basic motor speed in 20 adults with NF1 (Zoller et al., 1997).

Intelligence in neurotypical controls seems to be strongly associated with neuropsychological functioning in cognitive domains such as attention (Diaz-asper et al., 2004). The distribution of the full-scale intelligence quotient (IQ) of children with NF1 is shifted downward, although the variability in cognitive ability is similar to the general population (Ottenhoff et al., 2020). In neurotypical adults, an association between a lower than average IQ and reduced attention has been demonstrated (Diaz-asper et al., 2004), and reduced intellectual functioning correlated with reduced executive functioning (Arffa, 2007). In contrast, previous studies found no association between intelligence and attention in children with NF1 (Hyman et al., 2005) nor between attention and motor learning problems in adults with NF1 (Hyman, Shores and North, 2005; Zimerman et al., 2015).

Considering the high prevalence of attention and motor learning deficits clinically reported in children with NF1, and the limited and inconsistent findings in adults with NF1, further clarification of the presence or absence of these deficits in adults with NF1 is important. Hence, we examined attention, including alertness and sustained attention, and motor skill learning in adults with NF1 compared to neurotypical controls. We made use of standardized measures that examined alertness and sustained attention (De Sonneville, 2014). These measures have frequently been used in studying attention deficits in various disorders, including ADHD and children with NF1 (De Sonneville, 2014). Additionally, motor skill learning was examined by the sequential visual isometric pinch task (SVIPT) (Coxon et al., 2014; Reis et al., 2009) and intellectual performance was examined by administering four subtests of the Wechsler Adult Intelligence Scale (WAIS-IV-NL).

## Methods and Materials

### Subjects

In this study, 32 NF1 patients and 32 controls between 18 and 56 years participated. NF1 patients were recruited from the ENCORE-NF1 expertise center for Neurodevelopmental Disorders at the Erasmus MC upon referral by their treating neurologist or through the Dutch NF patient association (NFVN). The patients had a genetic and/or a clinical diagnosis. Controls matched for age and gender were unaffected peers of the patients or recruited through online advertisements. According to the inclusion and exclusion criteria, the subjects in the control group had no current presence or history of neurological, psychiatric or medical disorders. The subjects in the NF1 group had no current presence or history of neurological or medical disorders other than NF1, or psychiatric disorders except for the comorbidity with ADHD based on clinical diagnosis. Furthermore, subjects with NF1 were excluded if the neurological illness influenced the function of the central nervous system or motor tract, or influenced the function of the peripheral nervous system involving the sensory or motor function of the hands. All subjects had no severe hearing problems and/or visual problems. All subjects were not taking medication at the time of the study (except for contraceptives, and methylphenidate [*n*_NF1_ = 1]). The Dutch Central Medical Ethics Committee of the Erasmus Medical Center Rotterdam approved the study (MEC-2017-029, NL59730.078.16), which was conducted in accordance with the Declaration of Helsinki (2013). All subjects gave their written informed consent.

### Procedure

The subjects were asked to abstain from alcohol and caffeinated beverages 24 hours before the start of the measurements. All subjects completed the tasks in the laboratory between 01:00 PM and 04:00 PM after having a light lunch. The tasks examined intellectual performance, alertness, sustained attention, and motor skill learning.

#### Intellectual performance

Intellectual performance was examined by administering four subtests of the Wechsler Adult Intelligence Scale (WAIS-IV-NL). The tests included block design, matrix reasoning, vocabulary, and similarities. This selection has a high correlation with full-scale IQ score (Van Ool et al., 2018). Verbal intelligence quotient (VIQ) was estimated based on the subtests vocabulary and similarities. Performance intelligence quotient (PIQ) was estimated based on the subtests block design and matrix reasoning (De Sonneville, 2014). Furthermore, the level of education of the subjects was coded using the international standard classification of education (Schneider, 2013) varying from “early childhood education” (0) to “doctoral or equivalent” (8).

#### Alertness

Alertness is the reaction time to a stimulus without any preparatory cue, and reflects the intensity of attention (Sturm & Willmes, 2001). We measured alertness with the baseline speed task (BS) from the Amsterdam Neuropsychological Tasks (ANT) (De Sonneville, 2014), which is a standardized visual reaction-time task. The ANT has proven to have sufficient psychometric properties, such as validity and test-retest reliability (De Sonneville, 2014). Subjects were in front of a monitor, while holding their index fingers on both the mouse buttons. The subjects had to look at a cross in the middle of the screen. They had to press the mouse button as quickly as possible when the cross changed into a square with a randomized inter-stimulus time interval of 500 to 2500 ms. We measured the reaction time in ms and the change over time in reaction time, that is, stability of the reaction time.

#### Sustained attention

Sustained attention is the ability to focus for a longer period of time on an unpredictable and changing stimulus. We measured sustained attention with the standardized ANT visual sustained attention dots (SAD) task (De Sonneville, 2014). The subjects had to press “YES” if a pattern with four dots (target) was displayed, or they had to press “NO” if a pattern with three or five dots (non-targets) was displayed. Misses and false alarms were followed by an auditory feedback signal. Dot patterns were presented in 50 series of 12 trials with a post response interval of 250 ms. The duration of the task was between 12 and 15 minutes depending on the reaction time of the subjects. The task requires to remain focus over a longer period of time. Therefore, the actual performance can be assessed with the so called time-on-task (TOT) effects (Marchetta et al., 2008; Tucha et al., 2017; van der Meere & Sergeant, 1988). We measured the change in performance over time (Tucha et al., 2017). Therefore, we computed five consecutive periods for quantification of reaction time in seconds per series and number of misses; each consisted of 10 series of 12 trials.

#### Motor skill learning

**Figure 1.**
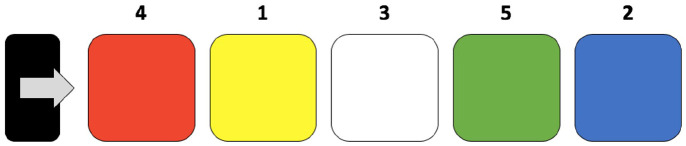
Schematic view of the motor skill learning task. The sequential visual isometric pinch task (SVIPT) was displayed on the monitor consisting of colored targets from left to right. Subjects had to move the cursor (arrow) back and forth from the home-box (black rectangle) to the targets in a predetermined order (1-2-3-4-5) by squeezing the transducer.

Motor skill learning was measured by the sequential visual isometric pinch task (SVIPT) (Coxon et al., 2014; Reis et al., 2009). The paradigm is a custom-built in a graphical user interface (GUI) in MatLab (MathWorks). The SVIPT is easy to understand, although it is challenging to perform as it shows improvements over 5 days in controls (Reis et al., 2009). This will prevent a ceiling effect in the control group and could be a sensitive task to detect differences between NF1 patients and a control group. Subjects were seated in front of a monitor, while holding a force transducer in their non-dominant hand. The SVIPT was displayed on the monitor consisting of colored targets from left to right (Figure 1). Subjects had to move the cursor between these targets in a predetermined order by squeezing the transducer. The cursor had to be on a target on each beat of a metronome of 1.67 Hz. Logarithmic transformation was applied to the force, to scale cursor movement according to Coxon et al. (Coxon et al., 2014). The farthest target was set at 45% of the maximum force. A total number of 12 blocks consisting of 15 trials were presented. The duration of the task was between 25 and 30 minutes. Visual feedback was displayed at the end of each block. We measured the change of performance over time by computing the slope of the reaction time and the error rate.

### Data Analysis and Statistics

Statistical analyses were performed in IBM Statistics SPSS (version 25). Nonparametric statistical tests were performed when assumptions for parametric statistics were violated. Demographics were compared between controls and NF1 patients with Chi square test, independent *t*-tests or Mann–Whitney *U*-tests. Reaction time and stability on the BS task were analyzed with independent *t*-tests using *z*-scores. Repeated measures ANOVAs were performed to analyze potential differences in reaction time and number of misses on the SAD between controls and NF1 patients over the five consecutive periods in time. Independent *t*-tests were performed to analyze the slope of reaction time and error rate on the SVIPT between controls and patients as quantified based on the 12 consecutive blocks in time. Statistical outliers were analyzed and removed if the value exceeded three standard deviations from the mean. Correlations were tested between outcome parameters computing Pearson correlation coefficients within the NF1 group. A *p*-value of *p* < .05 was considered to indicate a significant difference. The *p*-values were corrected for multiple testing with the Bonferroni correction.

### Data Availability

Data are available from the corresponding author on reasonable request.

## Results

We measured 64 participants (*n*_control_ = 32 *n*_NF1_ = 32). After the measurements, exclusion of participants was necessary due to technical problems during the attention tasks (*n*_control_ = 2; *n*_NF1_ = 1), insufficient knowledge of the Dutch language (*n*_control_ = 1), IQ recently tested, but not available (*n*_NF1_ = 1), and withdrawal during the motor learning task (*n*_NF1_ = 1). Age (*M*_control_ = 35.4 ± 11.0, *M*_NF1_ = 30.9 ± 12.0) and gender were not different between the groups (*t*_age_ [57] = 1.08, *p* = .28; χ^2^_gender_ = 0.167, *p* = .68). Educational attainment was significantly lower in the NF1 group than in the control group (*U*_ISCED_ = 303, *p* = .006) (Table 1).

**Table 1.** Demographics, intellectual performance, attention, and motor learning parameters (mean ± SD) of the NF1 group and the control group separately.

	NF1 group (*n* = 32)	Control group (*n* = 32)
Demographics		
°Age in years	30.9 ± 12.0	35.4 ± 11.0
°Gender: male in % (*#*)	41 (13)	50 (16)
°Educational attainment, median (range)*	4 (1–6)	5 (2–6)
Intellectual performance		
°Verbal IQ*	85 ± 16.6	99 ± 12.9
°°Similarities*	7.2 ± 3.1	9.4 ± 2.8
°°Vocabulary*	7.5 ± 2.8	10.2 ± 2.4
°Performance IQ*	87 ± 15.3	98 ± 19.6
°°Block design*	6.7 ± 2.8	9.4 ± 3.6
°°Matrix reasoning	9.0 ± 2.9	9.7 ± 3.4
Alertness		
°BS reaction time (ms)	282 ± 40.3	272 ± 26.7
°BS reaction time (*z*-score)	0.2 ± 1.1	−0.03 ± 0.8
Sustained attention		
°SAD reaction time (s per series)	9.1 ± 2.2	8.5 ± 1.6
°SAD variability in time (speed, s per series)	1.2 ± 0.7	1.0 ± 0.4
°Number of misses (# per series), median (range)	15 (2–96)	15 (2–74)
Motor learning		
°Reaction time (slope)*	−0.8 ± 0.9	−1.2 ± 0.7
°Error rate (slope)	−1.7 ± 1.1	−2.1 ± 1.0

* Significantly different between patients and controls (*p*-value < .05).

# Number of subjects; BS, baseline speed task; SAD, sustained attention dots task; series, consists of 12 trials with the representation of a dot pattern (in total 50 series of 12 trials).

### Intellectual Performance

Verbal IQ (*M*_control_ = 99 ± 12.9, M_NF1_ = 85 ± 16.6) and performance IQ (*M*_control_ = 98 ± 19.6, *M*_NF1_ = 87 ± 15.3) were significantly lower in the NF1 group than in the control group (Table 1; *t*_VIQ_[59] = 3.66, *p* = .001, *t*_PIQ_[60] = 2.42, *p* = .018). These results indicate a lower intellectual performance in adults with NF1 than controls. Furthermore, the scores on the subtests of the WAIS-IV-NL were all significantly lower in the NF1 group than in the control group (Table 1; *t*_block design_[61] = 3.53, *p* = .001, *t*_similarities_[60] = 2.94, *p* = .005, *t*_vocabulary_[60] = 4.17, *p* < .001), except for the subtest matrix reasoning. The scores on the subtest matrix reasoning were the same for both groups (*t*_matrix_[61] = −0.82, *p* = .41) (Table 1).

### Alertness

There were no significant differences in mean reaction time in ms (*M*_control_ = 272 ± 26.7, *M*_NF1_ = 282 ± 40.3) (*t_z_*_-score_[59] = −0.99, *p* = .33) or the stability of reaction time (U_RT_ = 472, *p* = .92) during the BS task between NF1 patients and controls (Table 1). Therefore, alertness tested with the BS task was not different between the groups.

### Sustained Attention

**Figure 2.**
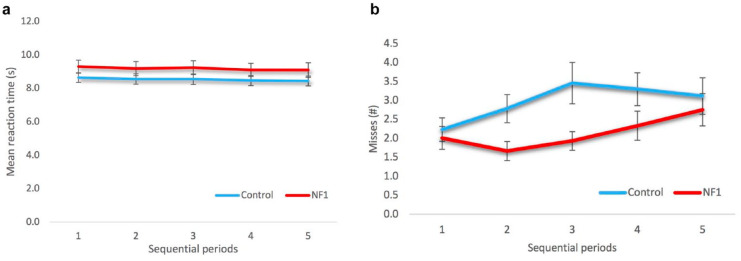
Sustained attention parameters on the sustained attention dots task (SAD) of the NF1 and control group: (a) The mean series reaction time in seconds ± SEM. There was no significant difference in mean reaction time between the groups (*F*[2.29, 141.8] = 0.85, *p* = .44), (b) The mean number of misses ± SEM. There was a nominally significant difference in the number of misses between the groups (*F*[1, 60] = 3.77, *p* = .057). There was a significant main effect over the five consecutive periods in time for the number of misses (*F*[4, 240] = 3.73, *p* = .006). There were no significant interaction effects.

Mean reaction time during the SAD task in seconds per series (*M*_control_ = 8.5 ± 1.6, *M*_NF1_ = 9.1 ± 2.2) was the same for both groups (*U*_RT_ = 385, *p* = .34) as well as the variability in time (speed) (*U*_speed_ = 383, *p* = .33). Therefore, sustained attention measured with the SAD task was similar for both groups. Mean reaction time during the SAD task did also not differ over the five consecutive periods in time (TOT effects) (*F*_RT_[2.29, 141.8] = 0.85, *p* = .44) (Figure 2a). In addition, there was no significant interaction effect between group and consecutive periods. Overall, controls made more misses, but this was nominally significant (*F*_misses_[1, 60] = 3.77, *p* = .057). There was a significant main effect over the five consecutive periods over time for the number of misses: controls made more misses during period 2 and 3 than NF1 patients (*F*_misses_[4, 240] = 3.73, *p* = .006) (Figure 2b). There was no significant interaction effect between group and consecutive periods.

### Motor Skill Learning

**Figure 3.**
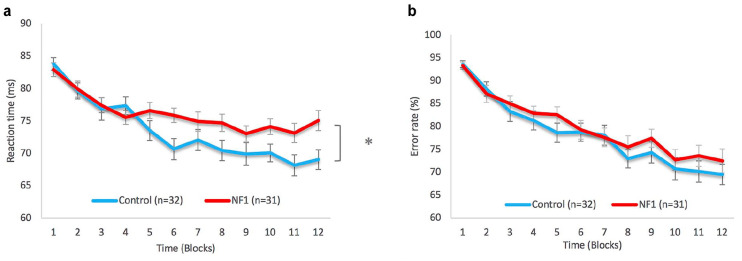
Motor learning parameters on the sequential visual isometric pinch task (SVIPT) of the NF1 and control group: (a) The mean reaction time ± SEM. There was a significant difference in the slope of the mean reaction time between the groups (*t*[60] = −2.20, *p* = .031) (b) The mean error rate ± SEM. There was no significant difference in the slope of the error rate between the groups (*t*[60] = −1.42, *p* = .16).

Although individuals with NF1 had a similar reaction time as neurotypical controls at the first training sessions, controls had significantly shorter reaction times than adults with NF1 during the motor learning task (slope of the reaction time during the SVIPT [*t*_slopeRT_(60) = −2.20, *p* = .031]; Table 1; Figure 3a). However, there was no significant difference in the slope of the error rate during the task between the groups (*t*_error rate_[60] = −1.42, *p* = .16), indicating that NF1 patients and controls both learned the task equally well (Table 1; Figure 3b).

### Correlations

We did not find significant correlations within the NF1 group between estimated IQ and reaction time during the BS task (*z-score*) (*r*_VIQ_ = −0.061, *p* = .75, *r*_PIQ_ = −0.228, *p* = .23), during the SAD task (*r*_VIQ_ = −0.405, *p* = .02, *r*_PIQ_ = −0.372, *p* = .04), or during the motor learning task (*r*_VIQ_ = −0.224, *p* = .24, *r*_PIQ_ = −0.072, *p* = .71). There were no significant correlations between estimated IQ and the error rate during motor learning (*r*_VIQ_ = −0.332, *p* = .07, *r*_PIQ_ = −0.308, *p* = .10). Furthermore, there were also no significant correlations between reaction time during the motor learning task and reaction time during the BS task (*z-score*) (*r*_BS_ = 0.108, *p* = .51) or during the SAD task (*r*_SAD_ = 0.03, *p* = .87). The *p*-values were corrected for multiple testing with the Bonferroni correction (α of .05 adjusted for 10 comparisons, *p* < .01).

## Discussion

Attentional and motor learning problems have been frequently observed in children with NF1 (Hyman et al., 2005), but less is known of the prevalence of these problems in adults with NF1. Based on the previous studies, we hypothesized that we would observe reduced alertness and sustained attention, as well as reduced motor learning in adults with NF1. However, these attention measures and this motor skill learning task did not provide convincing evidence for attention and motor learning problems in adults with NF1, although controls reached a faster reaction time compared to adults with NF1 in the motor learning task.

### Attention

Although attention is the most frequently affected ability in children with NF1 (Lehtonen et al., 2013), our findings did not provide evidence for the presence of attention deficits in adults with NF1. The absence of a difference in performance in the alertness and sustained attention tasks in adults with NF1 is in contrast to previous studies in children with NF1 (Isenberg et al., 2013; Michael et al., 2014; Payne et al., 2019; Torres Nupan et al., 2017). In two NF1 studies, the same measure of alertness showed diminished alertness in children with NF1 compared to controls (Huijbregts et al., 2011; Rowbotham et al., 2009). Furthermore, sustained attention was affected in 63% of children with NF1 (Hyman et al., 2005). The first reason for the lack of attention differences between adults with NF1 and controls could be due to developmental changes from childhood to adulthood (Descheemaeker et al., 2013; Michael et al., 2014). The delay in the development of attention components (Stuss, 1992) could reflect the attention deficits mainly seen in children with NF1 (Michael et al., 2014). It would be interesting to take the maturation process into consideration in future prospective longitudinal studies. The second reason could be the low incidence of clinically diagnosed ADHD in our sample (*n* = 1), which could indicate a potential recruitment bias. Mautner et al., (Mautner et al., 2015) showed that comorbidity of ADHD symptoms in NF1 patients persists during adulthood. However, attention problems should also be present in NF1 patients without ADHD symptoms according to Ribeiro et al. (2014). They showed that attention deficits are linked to a specific increase in the amplitude of alpha oscillations, which have been observed in children with NF1 without ADHD at rest and during visual stimulation (Ribeiro et al., 2014). Interestingly, this increased alpha was associated with a similar performance in children with NF1 and controls during a visual detection task, indicating that the aberrant alpha rhythm might still be functional. Therefore, it would be interesting to study alpha oscillations related to attention in adults with NF1 in future research. Finally, the reason for the lack of attention deficits in adults could be the result of using only quantitative performance-based measures of attention. Although these measures are objective and have been used in previous NF1 studies including measures of alertness and sustained attention (Isenberg et al., 2013; Michael et al., 2014; Payne et al., 2019; Torres Nupan et al., 2017), Biotteau et al., (2021) advised the use of both observer-rated questionnaires and performance based assessments.

Despite the fact that we did not see attention deficits in our NF1 cohort, it is known that adults with NF1 experience attention problems in their daily lives that affects their quality of life (Gutmann et al., 2017). In addition, learning disabilities and attention problems could predict problems in mental health in NF1 adults (Crawford et al., 2015). In the present study, attention deficits were not observed in an experimental setting, but keeping attention levels high, could be associated with increased fatigue in NF1 patients. Rietman et al., (2018) noted that fatigue has a large effect on the daily life of NF1 adult patients and that it also limited the coping skills of patients. Although patients did not express fatigue during the measurements or at the final evaluation in the present study, we still recommend including an objective measure of fatigue in the future studies. Additionally, our patients were highly motivated, which could have contributed to the significantly lower number of misses over time during the SAD task in the NF1 patients than in controls.

### Motor Learning

The observed overall slower reaction times in the NF1 group on the motor learning task is consistent with previous studies suggesting a slower information processing overall in NF1 (Huijbregts et al., 2011; Michael et al., 2014; Rowbotham et al., 2009). However, one study in adults with NF1 showed no specific problems in basic motor speed measured with a finger tapping test in 30 adults with NF1 (Zoller et al., 1997). Another explanation for the slower reaction time may be due to reduced maximal voluntary muscle force in NF1 (Souza et al., 2009). NF1 patients are known to have reduced maximum voluntary muscle strength (Souza et al., 2009) required to successfully perform the motor learning task by reaching the most distal target (Coxon et al., 2014). However, in our study, the most distal target was set to (only) 45% of their individual maximal muscle force needed to reach all the targets. Since the accuracy on the motor learning task was not significantly different between groups, potential reduced muscle force in adults with NF1 was unlikely to affect the performance on the motor learning task.

Our findings suggest that adults with NF1 and controls performed similarly on the motor learning task. This finding is in contrast to a previous study in 9 adults with NF1 that assessed a similar motor learning task over five consecutive days (Zimerman et al., 2015). That study indicated a relative inability to perform the motor learning task as well as controls, which was already evident at the first day of training. This difference was not observed in our study using the SVIPT, even though the duration of the measurement was three times longer in our study to make the task more challenging to perform and more sensitive to detect differences (Reis et al., 2009).

### Intellectual Performance

We found no association between intelligence and attention and motor learning problems in NF1 patients, which is consistent with previous studies in children and adults (Hyman, Shores and North, 2005; Zimerman et al., 2015). The estimated verbal and performance intelligence score of the NF1 patients are in line with previous studies (Hyman et al., 2005; Krab, de Goede-Bolder, et al., 2008; Ottenhoff et al., 2020). Ottenhoff et al., (2020) showed in 497 children with NF1 significantly lower IQ-scores, whereas the variability in IQ was similar to the general population. In our study with adults, most subscale IQ scores were significantly lower in NF1 patients than in controls, although patients had no diminished performance in matrix reasoning. Matrix reasoning measures visual-spatial functioning similar to the subtest block design, but is, in contrast with block design, independent of a time constraint. Subscale scores on matrix reasoning have not yet been described in NF1 patients. Possibly, deficits in processing speed were not addressed with matrix reasoning (Gorlyn et al., 2006).

### Strengths and Limitations

This study has three key strengths: the use of a relatively large sample size, the use of a representative NF1 sample, and the use of standardized test measures to assess attention. A large sample size might help to avoid bias in recruitment of patients. It is important to note that patients were free of any psychoactive medication, except for one patient receiving methylphenidate, that could affect the outcome measures. Additionally, patients were not receiving mental health care. The estimated IQ of adults with NF1 in our study suggests that our sample was cognitively affected in the same way as overall present in the NF1 population. Furthermore, it could be that the NF1 samples in previous studies were not representative for the population due to specific recruitment of only patients with academic problems (Heimgartner et al., 2019) or only patients with high education (Descheemaeker et al., 2013; Zimerman et al., 2015). Although educational attainment was significantly different between the groups, it did not predict the outcome measures in the present study as expected. Furthermore, age and gender were similar for the NF1 and patient group. Last, in the present study we used standardized measures of alertness and sustained attention frequently used in studies to measure attention deficits in various disorders (De Sonneville, 2014).

## Conclusion

To conclude, the present study shows a similar performance on attention and motor learning tasks in a representative NF1 adult sample in an experimental design, despite potential problems in these cognitive domains seen in the NF1 population. Overall, our NF1 patients seemed highly motivated to perform the tasks. Their similar performance on these tasks compared to controls may reflect this and may be related to the increased fatigue or other associated complaints in NF1 patients in their daily life. Research into attention in adults with NF1 has important clinical implications to determine treatment focus. It would be interesting to validate our findings by performing a prospective longitudinal study controlling for both the maturation process from childhood to adulthood and the heterogeneous cognitive phenotype.
